# Sepsis-induced cardiac dysfunction and β-adrenergic blockade therapy for sepsis

**DOI:** 10.1186/s40560-017-0215-2

**Published:** 2017-03-03

**Authors:** Takeshi Suzuki, Yuta Suzuki, Jun Okuda, Takuya Kurazumi, Tomohiro Suhara, Tomomi Ueda, Hiromasa Nagata, Hiroshi Morisaki

**Affiliations:** Department of Anesthesiology and General Intensive Care Unit, Keio University School of Medicine, 35 Shinanomachi, Shinjuku-ku, Tokyo 160-8582 Japan

**Keywords:** Sepsis, Sepsis-induced cardiac dysfunction, β-adrenergic blockade therapy

## Abstract

Despite recent advances in medical care, mortality due to sepsis, defined as life-threatening organ dysfunction caused by a dysregulated host response to infection, remains high. Fluid resuscitation and vasopressors are the first-line treatment for sepsis in order to optimize hemodynamic instability caused by vasodilation and increased vascular permeability. However, these therapies, aimed at maintaining blood pressure and blood flow to vital organs, could have deleterious cardiac effects, as cardiomyocyte damage occurs in the early stages of sepsis. Recent experimental and clinical studies have demonstrated that a number of factors contribute to sepsis-induced cardiac dysfunction and the degree of cardiac dysfunction is one of the major prognostic factors of sepsis. Therefore, strategies to prevent further cardiomyocyte damage could be of crucial importance in improving the outcome of sepsis.

Among many factors causing sepsis-induced cardiac dysfunction, sympathetic nerve overstimulation, due to endogenous elevated catecholamine levels and exogenous catecholamine administration, is thought to play a major role. β-adrenergic blockade therapy is widely used for ischemic heart disease and chronic heart failure and in the prevention of cardiovascular events in high-risk perioperative patients undergoing major surgery. It has also been shown to restore cardiac function in experimental septic animal models. In a single-center randomized controlled trial, esmolol infusion in patients with septic shock with persistent tachycardia reduced the 28-day mortality. Furthermore, it is likely that β-adrenergic blockade therapy may result in further beneficial effects in patients with sepsis, such as the reduction of inflammatory cytokine production, suppression of hypermetabolic status, maintenance of glucose homeostasis, and improvement of coagulation disorders.

Recent accumulating evidence suggests that β-adrenergic blockade could be an attractive therapy to improve the prognosis of sepsis. We await a large multicenter randomized clinical trial to confirm the beneficial effects of β-adrenergic blockade therapy in sepsis, of which mortality is still high.

## Background

Sepsis, defined as a life-threatening organ dysfunction caused by a dysregulated host response to infection, according to the third international consensus definitions for sepsis and septic shock [1], is one of the leading causes of death in the intensive care unit (ICU), despite significant recent advances in intensive care medicine [2, 3]. It is estimated that from 56 to 91 per 100,000 adults experience severe sepsis and septic shock worldwide each year [4], and the mortality rates from septic shock, a refractory severe hypotensive state, have ranged from 40 to 50% over the past decades [5]. It has been estimated that worldwide, one patient dies due to sepsis every few seconds and sepsis-related mortality has exceeded mortality due to acute myocardial infarction. Therefore, improving the prognosis in patients with sepsis remains a challenging area for clinicians working in the ICU.

Although the hemodynamic response to sepsis has been characterized as a hyperdynamic state, typically characterized by an increased cardiac output due to fluid resuscitation and decreased systemic vascular resistance, cardiac dysfunction occurs during the early stages of sepsis [6]. On echocardiography examination, sepsis-induced cardiac dysfunction is identifiable as a reduction in stroke volume and ejection fraction [6, 7]. Many factors have been shown to contribute to sepsis-induced cardiac dysfunction [8], and adrenergic overstimulation may exacerbate myocardial dysfunction during sepsis [9, 10]. Over recent decades, a growing body of experimental and clinical studies has focused on the beneficial effects of β-adrenergic blocker therapy for treating sepsis [9, 11], suggesting that this may be a promising therapeutic intervention.

In this review article, we summarize the pathophysiology of sepsis-induced cardiac dysfunction and discuss the potentially therapeutic effects of β-adrenergic blockade on sepsis-induced cardiac dysfunction and other damaged organs during sepsis.

## Review

### Hemodynamic management in septic shock

Sepsis is characterized by a dysregulated systemic inflammatory response caused by infection, leading to multiple organ injury and shock [1, 12]. Many mediators, such as pro-inflammatory cytokines, including tumor necrosis factor-α (TNF-α) and interleukin (IL-1β), nitric oxide, and reactive oxygen species, have been shown to cause cardiac dysfunction, increased vascular permeability, and reduced peripheral vascular resistance [8, 13], which can induce hemodynamic instability and multiple organ injury.

In 2001, Rivers et al. reported the findings of a single-center trial and concluded that early goal-directed therapy (EGDT), targeting mean blood pressure over 65 mmHg and oxygen saturation of central venous blood (ScVO_2_) over 70% within 6 h of the onset of severe sepsis, significantly reduced mortality rates [14]. Although, recently, three multicenter randomized trials have demonstrated that EGDT did not improve the outcome in patients with severe sepsis [15–17], it is clear that stabilizing hemodynamics at the early stages of sepsis is crucial for the management of septic patients, as the degree of lactate clearance has been shown to reflect the prognosis in critically ill patients [18].

During the early stages of sepsis, particularly in patients with septic shock, the primary aim of treatment is optimization of the hemodynamic status by adequate fluid resuscitation and vasopressors, in order to meet the oxygen demands of peripheral tissues and prevent organ injury [19]. However, excessive fluid and adrenergic overstimulation could be detrimental to the heart, which has already sustained injury during the early stages of sepsis. Previous studies have demonstrated that the mortality rate of patients developing cardiac dysfunction during the early stages of sepsis was higher than that of patients without cardiac dysfunction [20, 21], which implies that reducing cardiomyocyte damage is a very important strategy in the management of patients with sepsis in order to improve the prognosis.

### Sepsis-induced cardiac dysfunction

Calvin et al. first described myocardial dysfunction in adequately volume-resuscitated patients with septic shock in 1981, reporting a reduced ejection fraction and enlarged end-diastolic volume index [22]. Packer et al. demonstrated that surviving patients with sepsis had a decreased ejection fraction and increased end-diastolic volume index, which recovered between 7 and 10 days after the onset of sepsis; however, non-survivors maintained a normal ejection fraction and end-diastolic volume [6, 23], suggesting that cardiac dysfunction in sepsis is a compensatory mechanism to confer a protective effect against myocardial dysfunction.

Experimental studies have also identified sepsis-induced morphological and functional damage to the heart. A study examining cardiac morphological changes evoked by cecum ligation and puncture (CLP)-induced abdominal peritonitis in a sheep model described damage to the mitochondrial structure and impaired microcirculation, due to myocardial and vascular endothelial cell edema [24], which could contribute to cardiac dysfunction during the early stages of sepsis. In an ex vivo study, evaluating cardiac function in the working heart model 24 h after CLP in a rat model, dP/dt max, an indicator of cardiac systolic function, cardiac work, and cardiac efficiency, was impaired in a CLP rat, compared with a sham rat [25]. These experimental studies demonstrated structural and functional cardiac injuries, even though cardiac function could be modulated by the change of preload and afterload in clinical situations.

Recent clinical studies evaluating cardiac function of patients with sepsis by echocardiography also showed a reduced ejection fraction, followed by both systolic and diastolic dysfunction [21, 26, 27]. However, a number of studies did not find an increased left ventricular end-diastolic volume index, which was shown in the previous study [28–30]. Furthermore, it has been reported that impaired ejection fraction was associated with a poor prognosis [21], contrary to an earlier study by Packer et al. [23], which found that a reduced ejection fraction was associated with improved outcome. While there are some discrepancies among studies regarding the association between reduced ejection fraction and prognosis, there is clear evidence of an association between sepsis-induced cardiac morphological changes and the resulting myocardial dysfunction, manifested as decreased contractility and impaired myocardial compliance [31]. This progressive dysfunction develops during the early stages of sepsis and can affect prognosis.

### Mechanisms of sepsis-induced cardiac dysfunction

**Fig. 1:**
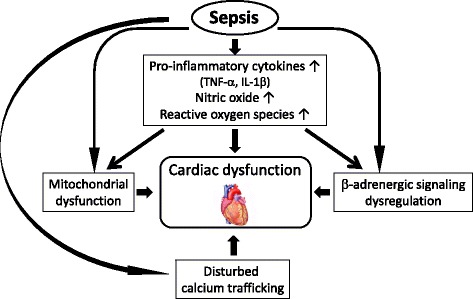
Mechanisms of cardiac dysfunction in sepsis. Many factors are associated with sepsis-induced cardiac dysfunction. *TNF-α* tumor necrosis factor-α, *IL-1β* interleukin-1β

Despite advances in our understanding of the pathophysiology of sepsis, the mechanisms of sepsis-induced cardiomyopathy have not been fully elucidated. Over recent decades, a number of experimental and clinical studies have suggested possible causative mechanisms for the progressive cardiac dysfunction observed in patients with sepsis (Fig. 1).

#### Disturbed coronary blood flow

In the 1970s, it was postulated that inadequate coronary blood flow, due to intravascular volume depletion, myocardial and endothelial cell edema, and vasodilation, was a major cause of sepsis-induced myocardial dysfunction [32, 33]. However, further human studies rejected the myocardial ischemia theory, demonstrating that coronary flow in patients with sepsis with cardiac dysfunction was comparable to, or greater, than coronary flow in controls [34, 35]. Furthermore, postmortem studies have found no myocardial necrosis in patients with septic shock [36]. While there may be changes in cardiac microcirculation in sepsis, caused by endothelial cell disruption and maldistribution of coronary blood flow, it is not obvious that myocardial ischemia contributes to the pathogenesis of sepsis-induced cardiomyopathy [8, 28].

#### Myocardial depressant factor and inflammatory cytokines

In an in vitro study conducted in 1985, Parrillo et al. found that serum from patients with sepsis depressed myocardial cell performance, unlike serum from critically ill patients without sepsis [37], and suggested that a circulating myocardial depressant factor (MDF) was the main cause of cardiac dysfunction in sepsis. Researchers subsequently investigated the molecular structure of MDF and concluded that MDF was likely to be an endotoxin and cell wall component of gram-negative bacteria. However, further studies revealed that the characteristics of inflammatory cytokines were comparable to those of MDF. Of these cytokines, tumor necrosis factor-α (TNF-α) and interleukin-1β (IL-1β), which are produced excessively at the early stages of sepsis, have been found to depress cardiac function synergistically [13, 38].

#### Nitric oxide and reactive oxygen species

TNF-α and IL-1β are major mediators causing myocardial dysfunction in sepsis. However, these cytokines have short half-lives, and studies have shown that their concentrations decrease in the early stages of sepsis. Therefore, other mediators, such as nitric oxide (NO) and reactive oxygen species (ROS), have been considered to be secondary effectors in sepsis-induced cardiac dysfunction [13, 39]. Excessive inducible NO synthase (iNOS), and specifically iNOS-2, induced in the myocardium by pro-inflammatory cytokines, results in a significant amount of NO production. This contributes to myocardial dysfunction through reduced sensitivity of myofibril response to calcium, inhibition of β-adrenergic signaling, downregulation of β-adrenergic receptor, and mitochondrial dysfunction [8, 28]. Peroxynitrite, produced by NO reaction with ROS, has a strong myocardial depressant effect with high cytotoxicity [40]. Reports indicate that NO and ROS cause mitochondrial dysfunction, as described in the following section.

#### Mitochondrial dysfunction

Mitochondrial dysfunction plays a key role in the pathogenesis of sepsis-induced cardiac dysfunction, leading to the so-called cytopathic hypoxia, which may contribute to multiple organ injury. NO and ROS contribute significantly to disturbed mitochondrial respiratory function, caused by inhibition of oxidative phosphorylation and adenosine triphosphate (ATP) production in the respiratory chain complex [8, 41]. Recent studies have demonstrated that mediators, including ROS and cytochrome C, released from mitochondria during cell death, could induce further inflammation [13].

#### β-Adrenergic signaling dysregulation

In patients with sepsis, adrenergic signaling dysregulation is associated with sepsis-induced cardiac dysfunction [8, 29, 42]. Despite increased circulating catecholamine levels, the contractile response of cardiomyocytes to catecholamine stimulation is blunted in patients with sepsis [8, 43]. Downregulation of β-adrenergic receptor and disturbance of β-adrenergic signaling are the key mechanism in this autonomic dysregulation [8, 29]. Sepsis may cause an increased activity of inhibitory G protein and a decreased accumulation of intracellular cyclic adenosine monophosphate (cAMP). Stimulatory G protein activity may be depressed through overproduction of inflammatory cytokines, leading to attenuation of β-adrenergic response to catecholamines [44, 45]. In sepsis, catecholamine overstimulation and elevated levels of NO may contribute to decreased β-adrenergic receptor density on the myocardial cell surface [46–48].

#### Calcium trafficking

Sepsis causes alterations of calcium trafficking at various sites, resulting in reduced cardiomyocyte contraction [8, 28]. Under physiological conditions, opening of L-type voltage-gated calcium channels on the cardiomyocyte sarcolemma, due to depolarization of the cardiomyocyte sarcolemma, causes calcium influx into the cardiomyocytes, leading to the release of calcium from the sarcoplasmic reticulum, through ryanodine receptors. This increase in intracellular calcium concentration plays a very important role in cardiac contraction. Reports indicate that sepsis is associated with the suppression of calcium current through L-type voltage-gated calcium channels [49, 50], decreased density of L-type calcium channels [49] and ryanodine receptors [51, 52], and a decrease in calcium uptake into the sarcoplasmic reticulum during the diastolic phase. Furthermore, calcium trafficking may contribute to mitochondrial dysfunction. Further studies are warranted to elucidate how these alterations in calcium homeostasis affect the long-term prognosis of patients with sepsis.

#### Cardiomyocyte apoptosis

In an ex vivo experimental model, it was found that inhibition of caspase activity, a key enzyme in apoptosis, reduced the depression of cardiac function. Therefore, it was postulated that apoptotic cardiomyocyte cell death was one of the mechanisms of sepsis-induced cardiac dysfunction [53]. However, cardiomyocyte apoptosis is unlikely to cause myocardial dysfunction in sepsis as postmortem examination of patients with sepsis has revealed negligible myocardial apoptosis [36].

### Protective effects of β-adrenergic blockers on sepsis-induced cardiac dysfunction

Although many studies have demonstrated that preventing cardiac injury is crucial to improving prognosis of septic patients [54, 55], effective treatment to attenuate cardiac dysfunction is not yet established. The mechanisms of sepsis-induced cardiac dysfunction have not been fully elucidated; nevertheless, some important factors contribute to the deterioration of cardiac dysfunction in the early stages of sepsis, as discussed above. Of these, catecholamine overstimulation plays a major role in sepsis-induced cardiac dysfunction [9, 56]. The elevated catecholamine level in sepsis can cause catecholamine-induced cardiomyopathy and cardiac damage by calcium overload, leading to cardiomyocyte necrosis. Furthermore, myocardial β-adrenergic receptor density is decreased and β-adrenergic stimulant signal transduction is impaired in sepsis [8, 29]. Therefore, prevention of further cardiomyocyte damage due to sympathetic nerve overstimulation could be a key component in the management of sepsis.

β-adrenergic blockers, first used for angina pectoris in the 1960s [57], have been widely prescribed for different diseases and conditions, such as ischemic heart disease and chronic heart failure [58], and perioperatively for patients with a high risk of cardiovascular events undergoing major surgery [59]. Berk et al. first reported the beneficial effects of β-adrenergic blockade therapy using an animal endotoxin shock model in the 1960s [60]; propranolol infusion reduced mortality from 78.2 to 19.4%. A further study, which included patients with refractory septic shock, reported a 27.3% mortality rate in patients treated with propranolol; this was low compared to the 30–40% mortality rate reported in recent studies. It is important to note that the management of patients with septic shock in these early studies was significantly different to the modern medical care available today [61]. Despite the beneficial effects in patients with septic shock, β-adrenergic blockade therapy in septic shock is not widely established, as results are conflicting. For example, a further study concluded that β-﻿adrenergic ﻿blockade in an endotoxin dog model worsened cardiac function [62]. Following the publication of this animal study, which disputed the beneficial effects of β-adrenergic blockade therapy, this field of research received scarce attention.

Approximately 35 years after Berk et al. described the possibility of the beneficial effects of β-adrenergic modulation in septic shock, the authors showed that β-adrenergic blockade therapy for sepsis attenuated sepsis-induced cardiac dysfunction, in an ex vivo experiment using a septic rat model [48]. We examined whether the selective β1-adrenergic blocker esmolol, continuously administered immediately after CLP was performed, could restore cardiac function in an isolated anterograde perfused heart preparation 24 h after esmolol infusion was started. During esmolol infusion, heart rate and mean blood pressure were significantly reduced with no lactate elevation compared to saline infusion. Cardiac output, cardiac work, and cardiac efficiency, an indicator of how efficiently the heart can use oxygen, were well maintained in hearts harvested from esmolol-treated rats compared with those harvested from non-treated rats. Furthermore, esmolol infusion reduced plasma TNF-α concentration and limited the reduction of β-adrenergic receptor density on cardiomyocytes. Although this study has not considered the effect of esmolol infusion on mortality, it was the first to demonstrate the beneficial effect of β-adrenergic blockade therapy on cardiomyocytes in sepsis. Further experimental studies confirmed the beneficial effects of selective β1-adrenergic blockade therapy in sepsis [63, 64], following our study, published in 2005.

The most serious concern regarding the clinical use of β-adrenergic blockade therapy in sepsis is the risk of reducing cardiac output and blood pressure, resulting in a further decrease in blood flow to major organs and potentially compromising organ function. Despite the risk of reduced organ blood flow due to the usage of β-adrenergic blockers, one clinical study demonstrated that esmolol infusion in patients with sepsis maintained hepatic blood flow, despite a 20% decrease in cardiac output [65]. Another retrospective study, examining the effect of enteral metoprolol on the hemodynamic state of patients with septic shock, showed that stroke volume was increased and cardiac output remained stable despite increases in the administered dose of noradrenaline and milrinone in some patients [66]. These results indicate that β-adrenergic blockade in patients with sepsis may be safe if adequate volume resuscitation therapy is performed.

Morelli et al. evaluated the beneficial effect of esmolol on septic shock patients in a single-center randomized controlled study [67]. In this study, 154 patients with septic shock, requiring noradrenaline infusion to maintain blood pressure and presenting with persistent tachycardia [>95 beats per minute (bpm)] after adequate volume resuscitation, were assigned to an esmolol infusion therapy group to decrease the heart rate to 80–94 bpm or to a saline infusion group. All patients in the esmolol group achieved the target heart rate of 80–94 bpm, which was the primary outcome. Furthermore, esmolol infusion increased the stroke volume index and reduced the fluid volume and norepinephrine dose to achieve a mean arterial pressure of 65–75 mmHg. Surprisingly, the 28-day mortality was significantly reduced from 80.5 to 49.4% in the esmolol group, without adverse events, compared with the control group. Despite the extremely high mortality in the control group and the widespread usage of levosimendan in both groups (49.4% in the esmolol group and 40.3% in the control group), this is the first clinical randomized controlled trial to show the beneficial effects of β-adrenergic blockade therapy in patients with septic shock.

Recently, an experimental study was conducted to identify the mechanisms underlying the beneficial effects of β-adrenergic blockade therapy in sepsis. Kimmoun et al. examined the effect of esmolol on cardiac and mesenteric vascular function in an ex vivo experiment, using a peritonitis-induced septic rat model [68]. Esmolol infusion counteracted the decreased cardiac contractility and the suppressed vasoreactivity to vasopressor treatment, induced by cecum ligation and puncture. Restored cardiac and vascular function through esmolol infusion was associated with decreased nuclear factor κB activation and reduced inducible nitrite oxide synthase expression, both at the cardiac and at the vessel level.

Further studies will be required to elucidate the effects of β-adrenergic blockade therapy in sepsis on cardiac function. The results of a multicenter controlled trial, evaluating the effect of β-adrenergic blockade therapy in a large number of patients with septic shock, are currently awaited.

### Beneficial effects of β-adrenergic blockade other than cardioprotective effects in sepsis

A growing body of research is focusing on the effect of β-adrenergic blockade therapy in sepsis [9, 69], specifically examining the beneficial effects other than those on the cardiovascular system. These are discussed in the following section.

#### Metabolic alterations

Sepsis is associated with an overall catabolic state, leading to hyperglycemia, enhanced protein and fatty breakdown, increased resting energy expenditure, negative nitrogen balance, and loss of lean body mass [70, 71]. This hypermetabolic state is predominantly caused by catecholamine overstimulation, particularly by β2-adrenergic stimulation [72, 73]. Thus, non-selective β-adrenergic blockade may counteract this hypermetabolic state associated with sepsis, contributing to the maintenance of glucose homeostasis, improvement of net nitrogen balance, and reserved muscle protein. In children with severe burns, characterized by a pathophysiology similar to that of septic shock, propranolol treatment reduced muscle protein catabolism and suppressed resting energy expenditure, leading to increased lean body mass. In septic rat models, propranolol infusion improved the nitrogen balance, possibly through a reduction of muscle proteolysis [74]. Considering the benefits of esmolol infusion in patients with burns, non-selective β-adrenergic blockade in patients with sepsis may have the same beneficial effects.

#### Cytokine production and immune modulation

In sepsis, the binding of lipopolysaccharides to toll-like receptor 4 promotes the translocation of the transcription factor NF-κB into nuclei, leading to a shower of cytokines. The increased levels of inflammatory cytokines further stimulate immunologically competent cells, contributing to a dysregulated hyper-inflammatory condition, with deleterious effects of activated neutrophils on different organs. Whether β-adrenergic blockade therapy in patients with sepsis has beneficial effects on the immune system requires further examination. However, it is well known that the β-adrenergic system is associated with immune system modulation [75]. Catecholamines have been shown to modulate the balance between pro-inflammatory and anti-inflammatory status through a β2-mediated pathway [76–78]. It has been reported that the pattern of cytokine production is strongly affected by the balance between CD4+ T-helper type 1 (Th1) and type 2 (Th2) cells. Th1 cell activation leads to activation of macrophages and natural killer T cells and production of pro-inflammatory cytokines, resulting in the promotion of cellular immunity. Conversely, Th2 cells inhibit macrophage activation, T cell proliferation, and pro-inflammatory cytokine production, through promotion of humoral immunity and production of anti-inflammatory cytokines [75]. Th1 cells, but not Th2 cells, have β2-adrenergic receptors on their surface. Stimulation of β2-adrenergic receptors suppresses Th1 cell activation, with a relative increase in Th2 cell response. Therefore, selective β1-adrenergic blockade could promote β2-adrenergic pathway activation, facilitating Th2 cell responses and contributing to the suppression of the pro-inflammatory status at the early stages of sepsis [9] and the activation of the anti-inflammatory pathway [79]. Conversely, β2-adrenergic blockade may enhance the inflammatory response, leading to pro-inflammatory cytokine production. The attenuation of the intense pro-inflammatory status at the early stages of sepsis, by selective β1-adrenergic blockade, may prevent the sequential immunosuppressive status.

In our study evaluating the effect of selective β1-adrenergic blockade on cardiac dysfunction in septic rat models, esmolol infusion significantly reduced plasma TNF-α concentration [48], and this may minimize cardiac dysfunction. A study by Hagiwara et al. demonstrated that a highly selective β1-adrenergic blocker, landiolol, decreased the levels of circulating cytokines, such as TNF-α, IL-6, and high-mobility group box 1, in an experimental septic model [63]. While the precise mechanism of β1-adrenergic blockade-mediated suppression of cytokine production was not elucidated in these studies, relative β2-adrenergic pathway activation may contribute to a reduction of pro-inflammatory cytokine production, as described above. Further studies are required to identify the mechanism by which selective β1-adrenergic blockade affects cytokine release.

In sepsis, it has been shown that lymphocyte apoptosis may be induced by a high inflammatory status, contributing to a worse prognosis [80]. In an experimental septic model, Hotchkiss et al. found splenocyte apoptosis in postpartum patients with septic shock [81] and demonstrated that inhibition of caspase, a key enzyme causing lymphocyte apoptosis, improved the prognosis, by preventing lymphocyte apoptosis [80]. Therefore, modulation of lymphocyte apoptosis could be an attractive therapeutic option to improve the prognosis of sepsis. One of the key pro-inflammatory cytokines in sepsis, TNF-α, can cause T lymphocyte apoptosis [82], and β2-adrenergic blockade has been reported to induce splenocyte apoptosis [83]. Therefore, through attenuation of TNF-α production and relative β2-adrenergic pathway stimulation, selective β1-adrenergic blockade may prevent lymphocyte apoptosis causing secondary infection and increased mortality. In our laboratory, the effect of selective β1-adrenergic blockade on splenocyte apoptosis has been examined in a septic mouse model. Esmolol treatment restored the number of normal T lymphocytes in the spleen, which was severely reduced 24 h after CLP, compared with the control group receiving a saline infusion. This finding supports the hypothesis that attenuation of lymphocyte apoptosis is one of the major mechanisms through which β1-adrenergic blockade has a positive effect in sepsis.

#### Coagulation disorder

Sepsis induces altered platelet function [84, 85], activation of the coagulation system, and suppression of fibrinolysis [9]. Increased levels of plasma tissue factor and von Willebrand factor amplify the coagulation cascade, leading to thrombin and fibrin formation [86]. Endothelial damage caused by thrombin formation further augments the coagulation cascade through more exposed tissue factor. Furthermore, impairment of the physiologic anticoagulation system occurs through downregulation of anticoagulant factors, such as tissue factor pathway inhibitor, antithrombin, and activated protein C, in sepsis [9]. Reports indicated that increased levels of TNF-α and IL-1β enhance the production of plasminogen activator inhibitor 1, leading to further impaired fibrinolysis [9]. A dysregulated coagulation system causes disseminated intravascular coagulation, leading to microcirculation disturbance and multiple organ injury.

Adrenergic pathways are associated with the coagulation system in different situations. Regarding platelet function, α2-adrenergic stimulation promotes platelet aggregation, while the β2-adrenergic pathway contributes to the suppression of platelet aggregation through cAMP stimulation [87]. β2-adrenergic stimulation promotes tissue plasminogen activator release, leading to enhanced fibrinolytic activity [88], while β1-adrenergic stimulation suppresses fibrinolysis through reduced prostacyclin synthesis [89].

Considering the association between the adrenergic pathway and the coagulation system described above, modulation of the β-adrenergic pathway could modify the hyper-coagulation status induced by sepsis. Regarding platelet function, β1-adrenergic blockade may reduce platelet activation through relative β2-adrenergic pathway activation. β1-adrenergic blockade could also enhance fibrinolysis through increased plasminogen activation and prostacyclin synthesis. Furthermore, reduction of pro-inflammatory cytokine production by β1-adrenergic blockade could reduce the increased plasminogen activator inhibitor 1 production, leading to improved fibrinolysis. There are few studies examining the beneficial effects of β1-adrenergic blockade on the disturbed coagulation system in sepsis, and this novel field should be examined in future studies.

### β-adrenergic blockade therapy for sepsis in the clinical situation

Although many beneficial effects of β-adrenergic blockade therapy in sepsis have been recently described, few studies have evaluated the effects of β-adrenergic blockade therapy on sepsis in clinical situations. Table 1 shows the summary of four clinical trials that examined the effects of β-﻿﻿adrenergic ﻿blockers in patients with sepsis. Only one randomized controlled trial evaluated the effects of β-adrenergic blockade therapy in septic patients; therefore, it is difficult to determine when and how β-adrenergic blockade therapy should be used in clinical practice. One of the major concerns regarding the use of β-adrenergic blockers in sepsis is the reduction of blood pressure and cardiac output, resulting in decreased blood flow to major organs, which can cause organ injury. However, in a number of studies, cardiac output was maintained and stroke volume index was increased, despite the reduction in heart rate [66, 67]. A further study, investigating the effects of esmolol infusion on hepatic and peripheral blood flow in sepsis, found that hepatic and peripheral blood flow did not change, despite reduced cardiac output [65]. Therefore, it is likely that in patients with sepsis, administration of β-﻿adrenergic﻿ blockers is relatively safe if patients have received adequate volume resuscitation. Sepsis-induced cardiac dysfunction develops in the early stages of sepsis; therefore, it seems reasonable to initiate β-adrenergic blockade therapy as early as possible after adequate volume resuscitation therapy, if persistent tachycardia does not improve. The duration of therapy and the target heart rate range are further important factors when administering β-adrenergic blockade therapy to septic patients. There are no studies investigating the optimum duration of β-adrenergic blockade therapy, which remains unknown. As the patient’s condition improves, the heart rate may return to baseline levels, before the onset of sepsis, without β-﻿adrenergic﻿ blocker therapy. In the four clinical trials [61, 65–67] presented in Table 1, β-﻿adrenergic ﻿blocker administration was adjusted to achieve a heart rate <95 bpm, and the heart rate was maintained between 80 and 95 bpm. Therefore, the optimum heart rate may be between 80 and 95 bpm.

**Table 1:** Summary of four clinical trials evaluating the effects of β-adrenergic blockade therapy in patients with sepsis

Reference number	Generic name of β-blocker	Method of administration	Dose of drug	Duration of therapy	Effects on hemodynamics	Mortality
61	Propranolol	Continuous iv infusion	5 mg for 2–3 h, followed by 5 mg for 6–12 h	8–15 h	HR↓ Cardiac output↓ Blood pressure↑ Urinary output↑	27.3% No control
65	Esmolol	Continuous iv infusion	6–22 mg/min (dose to reduce HR by 20%)	3 h	HR↓ Cardiac output↓ SVR→ Hepatic blood flow→	No data
66	Metoprolol with milrinone	Enteral administration	25–47.5 mg/day (target range of 65–95 bpm)	48 h	HR↓ Cardiac output→ Catecholamine dose↓ SVI↑	28-day mortality: 33%
67	Esmolol	Continuous iv infusion	Median dose of 100 mg/h (dose to maintain HR from 80 to 94 bpm)	Until ICU discharge or death	HR↓ Cardiac index→ Mean blood pressure→ Norepinephrine dose↓ SVI↑	28-day mortality: Control 80.5% Esmolol 49.4%

*bpm* beats per minute, *HR* heart rate, *ICU* intensive care unit, *iv* intravenous, *SVI* stroke volume index

β-adrenergic blockade therapy for patients with sepsis remains controversial due to limited evidence in the clinical context. It is important to consider potential adverse effects and pitfalls of β-﻿adrenergic ﻿blocker therapy before its use in patients with sepsis. As discussed above, the first adverse event to consider is the reduction of blood flow to major organs, due to decreased heart rate and cardiac output. Therefore, before administering β-blocker therapy, it is important to establish adequate volume resuscitation and the optimal dosage of norepinephrine, using the following parameters: diameter of the inferior vena cava evaluated by echocardiography, stroke volume variation, and systemic vascular resistance, which can be measured by arterial pressure-based cardiac output, and a central venous catheter. Interestingly, in the study by Morelli et al. evaluating the effect of β-blocker therapy following adequate volume resuscitation [67], mean arterial pressure was maintained, despite reduced norepinephrine and fluid requirements in the esmolol group. Furthermore, kidney function, evaluated by the estimated glomerular filtration rate, was maintained, and cardiac injury, assessed by troponin T and creatine kinase (CK)-MB, was reduced by esmolol administration. Taking into consideration that the heart rate was maintained between 80 and 94 bpm in the study by Morelli et al. [67], and mean heart rates were 78 and 90 bpm, respectively, in two recent clinical trials [65, 66], it could be unsafe to reduce the heart rate to <80 bpm. To achieve the beneficial effects of β-adrenergic blockade therapy in patients with sepsis, it appears that the heart rate should be maintained within a narrow range. A further concern is the harmful effect of β2 receptor blockade on respiratory function. However, the effect on respiratory function may be negligible due to the high β1 receptor selectivity of esmolol and landiolol.

## Conclusions

**Fig. 2:**
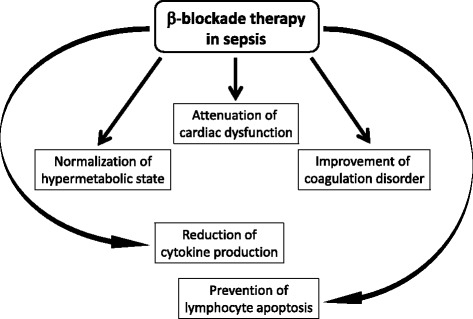
Beneficial effects of β-adrenergic﻿ blockade therapy for sepsis. β-adrenergic blockade therapy has various potential therapeutic effects in patients with sepsis

This review focuses on the mechanisms of sepsis-induced cardiac dysfunction and the beneficial effects of β-adrenergic blockade therapy, predominantly on the cardiovascular system and other organs (Fig. 2). Promising results are accruing and these show the beneficial effects of β-adrenergic blockade therapy in sepsis. β-adrenergic blocker therapy could be a promising novel therapeutic approach to modulate cardiovascular dysfunction, as well as metabolic and immune disorders and disorders of the coagulation system, as hyperactivation of the sympathetic nervous system could have deleterious effects on a wide range of organs. Experimental and clinical research is required to elucidate the β-adrenergic blocker therapy-mediated beneficial effects in sepsis, before β-adrenergic blocker therapy is widely used in clinical practice. It is our view that large multicenter randomized clinical trials could confirm the beneficial effects of β-adrenergic blockade therapy in patients with sepsis, improving the prognosis of sepsis which, to date, has a high mortality rate.

## Abbreviations

ATP: Adenosine triphosphate
cAMP: Cyclic adenosine monophosphate
CK: Creatine kinase
CLP: Cecum ligation and puncture
EGDT: Early goal-directed therapy
ICU: Intensive care unit
IL-1β: Interleukin-1β
iNOS: Inducible nitric oxide synthase
MDF: Myocardial depressant factor
NO: Nitric oxide
ROS: Reactive oxygen species
ScVO_2_: Oxygen saturation of central venous blood
Th1: CD4+ T-helper type 1
Th2: CD4+ T-helper type 2
TNF-α: Tumor necrosis factor-α
